# Ketogenic diet as a therapeutic intervention for obsessive-compulsive disorder: a case series of three patients

**DOI:** 10.3389/fnut.2025.1568076

**Published:** 2025-04-16

**Authors:** Aaron John MacDonald, Christopher Michael Palmer

**Affiliations:** ^1^McLean Hospital, Belmont, MA, United States; ^2^Harvard Medical School, Boston, MA, United States

**Keywords:** obsessive-compulsive disorder, ketogenic diet, metabolism, mitochondria, neuropsychiatric disorders, carnivore diet

## Abstract

**Introduction:**

The ketogenic diet is being explored as a therapeutic intervention for the treatment of neuropsychiatric disorders. Emerging research suggests that these conditions share common pathophysiologies, with the ketogenic diet showing promise in addressing these. This study reports three individuals who reduced their symptoms of obsessive-compulsive disorder (OCD) after adopting a ketogenic diet.

**Methods:**

Participants were recruited through personal and professional networks among the authors. Each patient was interviewed, and evidence of their mental health history was collected. Their OCD symptoms were retrospectively assessed before and after adopting the diet using the Yale-Brown Obsessive Compulsive Scale (Y-BOCS).

**Results:**

The three participants in this case series have all achieved remission of their symptoms and are medication-free. The diet implementation reduced their average Y-BOCS scores by 21 points, corresponding to a mean decrease of 90.5%. In all cases, deviations from the ketogenic diet resulted in a return of their symptoms.

**Conclusion:**

The ketogenic diet may be an effective treatment for obsessive-compulsive disorder. Its capacity to improve the metabolic dysfunction associated with OCD may target the underlying mechanisms of the disorder. Controlled clinical trials of the ketogenic diet as a treatment for OCD are warranted.

## Introduction

The ketogenic diet (KD) is gaining interest in psychiatry for its therapeutic potential. Limiting daily carbohydrate intake to 20–50 grams, the diet is moderate in protein and high in fat. Initially used by neurologists to treat epilepsy (1), the KD has proven effective even when medications fail (2). Its profound effects on brain metabolism in epilepsy have prompted research into its use for various neuropsychiatric conditions. Studies show symptom improvement in schizophrenia, bipolar disorder, Alzheimer’s disease, cognitive impairment, autism spectrum disorder, anxiety, and depression (3). However, research on the KD as a treatment for obsessive-compulsive disorder (OCD) is lacking.

Several transdiagnostic pathophysiologies, such as mitochondrial dysfunction, oxidative stress, inflammation, glucose hypometabolism, and glutamate/gamma-aminobutyric acid (GABA) imbalance, are thought to contribute to neuropsychiatric disorders, including OCD (3). Understanding the interplay between metabolism and mental illness may unveil novel treatment strategies.

Metabolic dysfunction is strongly implicated in OCD. Alterations in mitochondrial DNA copy number (mtDNAcn), a marker of mitochondrial dysfunction, are noted in OCD, controlling for factors like age and BMI (4). Oxidative stress is evident, with a meta-analysis revealing significantly elevated oxidant markers in individuals with OCD compared to controls (5). Inflammation is heightened in OCD-related neurocircuitry, including a 30.9–35.6% increase in the cortico-striato-thalamo-cortical (CSTC) circuit, 23.5% in the anterior cingulate cortex (ACC), and 22–29% in other gray matter areas (6). Glucose hypometabolism affects the insula, caudate nucleus, and middle temporal gyrus, while hypermetabolism characterizes the orbitofrontal region (7). An elevated glutamate/GABA ratio, linked to excessive neural activity, is also observed in the ACC (8) and CSTC (9) circuits. The KD addresses these disruptions, improving mitochondrial function (10), reducing oxidative stress (11), mitigating inflammation (12), normalizing glucose hypometabolism (13), and potentially restoring the balance between glutamate and GABA (14).

While OCD and epilepsy are distinct disorders, both are associated with several pathophysiologies which may be relevant to the potential therapeutic effects of the KD. Like OCD, epilepsy is also associated with metabolic dysfunction (15). While the exact mechanisms by which the KD helps manage epilepsy remain unclear, its therapeutic effects may be linked to addressing these metabolic abnormalities. Since OCD is also associated with similar metabolic dysfunctions, the KD may offer potential benefits for OCD and epilepsy through analogous pathophysiological mechanisms.

KDs have been shown to increase mitochondrial capacity and efficiency, as indicated by increased total adenosine triphosphate (ATP) production relative to oxygen gas consumption and hydrogen peroxide production (10). The KD has been shown to decrease oxidative stress, which can cause an excess of radical oxygen species (ROS) production in a pathological positive feedback loop (11). A meta-analysis of 44 randomized controlled trials found that the KD was effective in reducing biomarkers of inflammation such as tumor necrosis factor-alpha (TNF-*α*) and interleukin (IL)-6 (12). By inducing ketosis, the diet provides ketone bodies that have been shown to resolve glucose hypometabolism by providing an alternate fuel source to glucose (13). The KD lowers aspartate levels, inhibiting glutamate decarboxylase (16), leading to a rise in GABA production (14). This increase in GABA may help correct the glutamate/GABA imbalance, potentially restoring the brain’s balance between inhibition and excitation.

Given the metabolic abnormalities associated with OCD and the therapeutic effects of KD on these pathophysiologies, it is plausible that KD could serve as an effective treatment for OCD, especially if these metabolic disturbances play a role in the symptoms of OCD. This case series reports three patients who achieved remission from OCD using KD. To our knowledge, this represents the first report documenting the effects of the KD on OCD. The study assesses KD’s impact on OCD, outlines its risks and limitations, and provides recommendations for future research.

## Methods

### Enrollment

Participants in the study were selected using a combination of professional connections and public outreach. One case came to attention through CP’s private practice, another was identified due to their outspoken presence on social media, and the third had publicly shared their experience online. All candidates openly shared that the KD helped their OCD. Each patient was interviewed, after which they were sent two Yale-Brown Obsessive Compulsive Scale (Y-BOCS) assessments to complete and return via email. One assessment was used for a retrospective score analysis, evaluating the severity of OCD before starting the KD. The second assessment was of their current OCD symptoms. Remission was defined as a Y-BOCS score decrease to 14 or fewer points post-treatment (17). The participants were asked to provide either medical documentation confirming their diagnosis and treatment history or corroborating statements from family members.

### Ethics

Written informed consent for the publication of this case series was obtained from all participants and, where applicable, their corroborators. Each patient received a copy of their case summary, with their report’s accuracy confirmed before obtaining their consent for submission. Pseudo-initials were used to protect their identity.

## Case 1

Patient BB, a 22-year-old male currently enrolled as an undergraduate at Harvard College, was diagnosed with OCD and generalized anxiety disorder (GAD) at age 4. His symptoms began at 18 months, with his parents reporting consistent object alignment. His orderliness persisted through playdates, where he often cleaned and organized his friends’ toys. By 3 years of age, daycare staff noted the presence of excessive handwashing. At 4 years old, he exhibited symmetry-seeking compulsions; if he ever spun in a circle, he turned in the opposite direction to “balance things out.” When giving his parents hugs or kisses, he would do so equally on both sides of their person, often in pairs. In art classes, he exclusively produced symmetrical artwork.

At age 9, his parents removed grains from his diet to address concerns about his body weight, unexpectedly noticing a dramatic reduction in his OCD symptoms. They continued eliminating specific foods between ages 9 and 13 to observe further effects. By age 15, the patient found that a KD was most effective in managing his symptoms. Within 2 weeks of initiating the KD, BB experienced a complete cessation of ritualistic behaviors, with his symptoms reduced to occasional intrusive thoughts. While symptoms occasionally resurfaced during periods of high stress, he used his dietary regimen to manage them. At age 17, he began cognitive behavioral therapy (CBT) focused on cognitive distortions to manage these thoughts.

Deviations from the KD have resulted in the return of his symptoms. He once paused the regimen at age 16 while on vacation to indulge in high-carbohydrate foods. In the subsequent days, he started collecting shampoo and conditioner bottles from his hotel room and stayed up late into the night, organizing them into neat rows. He has since been compliant with the diet, only deviating from it with occasional high-carbohydrate snacks.

The patient’s Y-BOCS score improved from 27 to 4 currently. Today, his diet includes meat, eggs, dairy, vegetables, and nuts. He occasionally eats low glycemic index berries, such as raspberries and blackberries. He estimates that he can deviate from his diet once or twice a month without a return of OCD symptoms. In addition to reductions in his symptoms of OCD, he reports having decreases in symptoms of eczema which had been present since infancy. To ensure that he remains in ketosis, he occasionally tests his ketone-to-glucose ratio using at-home blood ketone and glucose monitors, though he has measured it daily in the past.

### Patient perspective

“The ketogenic diet was transformative for resolving my OCD, mood disorders, and focus issues. Without making the changes to my diet that I did, I would not have had the mental wherewithal to perform well enough in high school to get into Harvard, much less college.”

## Case 2

Patient NM, a 35-year-old female, developed symptoms of OCD at age 16 following a traumatic mass shooting near her school. This incident triggered intrusive thoughts characterized by a fear of losing control and causing harm, leading to obsessive efforts to prevent “something bad” from happening. In response, she disposed of all scarves, belts, and sharp objects, fearing she might act on these thoughts. In fear of harming her family, NM began isolating herself from them, staying up all night and sleeping during the day to avoid contact with her siblings and parents. She frequently contemplated running away from home and occasionally engaged in non-suicidal self-injurious behaviors directed at her head to alleviate intrusive thoughts.

When considering which college to attend, NM decided to go to the college that was the furthest away from her home to protect her family from any harm she feared she might cause. After moving to college, her symptoms persisted, now directed toward her new roommates. She spent extensive periods alone, taking long walks to avoid being around others. On one occasion, she was out until midnight, unable to return to her residence. Her family arranged for her to see a psychologist, who diagnosed her with OCD when she was 20 years old.

After a few therapy sessions, she decided to halt her education temporarily and moved home to be with her family to continue treatment. The patient relied on her mother for transportation to therapy due to fears of harming others while driving. She began CBT, centered around exposure and response prevention, to help her process intrusive thoughts without avoidance. While this helped manage her symptoms, her intrusive thoughts and compulsion to isolate herself continued to be burdensome. NM was then evaluated by a psychiatrist who recommended she take an SSRI, but she declined due to concerns over potential health impacts. Continued CBT helped reduce her avoidance behaviors enough for her to return to college and finish her degree.

Following college, however, her OCD returned to “full force” after moving in with her partner at 31 years of age. She once again experienced intrusive thoughts and a strong urge to isolate herself. During this time, she also noticed a blueish discoloration on her abdomen while showering. Alarmed by these developments, she became concerned about a general decline in her health and decided to intervene with a dietary regimen: the KD. Her diet consisted of meat, eggs, dairy, and low-carbohydrate fruits and vegetables.

NM reports a substantial reduction in intrusive thoughts and avoidance behaviors within two weeks of starting the KD. Encouraged by this improvement, she has adhered to the diet for three years and remains nearly symptom-free. Her Y-BOCS score decreased from 22 to 3 after initiating the diet. Reintroducing high-carbohydrate foods triggers symptom recurrence, which she describes as feeling like “swimming in a lake as a thunderstorm approaches.” To monitor her remission, NM periodically measures blood ketone levels and is currently experimenting with the gradual reintroduction of more foods into her diet. When starting the KD, she kept a log of blood ketone levels, measuring them 51 times over 1.5 years, with most values recorded weekly and ranging between 0.4 mmol/L and 3.7 mmol/L. She did not observe a direct correlation between the depth of ketosis and symptom reduction, though deviations from the diet result in symptom recurrence, which she attributes to halting ketosis.

### Patient perspective

“I used to tell myself in the depths of OCD, ‘The only way out is death,’ as a kind of mantra to put things into perspective. I’m happy to say I found another way. It would make me really happy if others knew about ketosis as a way to end their suffering.”

## Case 3

Patient GH is a 47-year-old female who was diagnosed with postpartum depression (PPD) at 28, bipolar disorder at age 34, OCD at age 35, and Hashimoto’s thyroiditis at age 44. She has a history of streptococcal infection and Epstein–Barr virus (EBV) infection, with uncertain timing. She tested positive for EBV antibodies at age 47 after her mother’s death from EBV-associated lymphoma and recalls having strep throat in middle or high school.

After the birth of her son at age 28, she developed symptoms of OCD, including orderliness, perfectionism, and a strict adherence to rules. While the formal diagnosis of OCD was made at age 35, these symptoms began coinciding with her PPD. She often stayed up as late as 4:00 am cleaning and organizing her home, refusing to sleep until it was exactly as she liked it. As a scout leader, she worked tirelessly writing emails to other scout leaders that sometimes exceeded four typed pages. Following her time volunteering with Scouts, GH attempted to work as a graphic designer, a county park educator, and in retail between the ages of 28–33; however, her need for perfectionism and rule adherence ultimately prevented her from succeeding and persisting in these roles.

GH’s OCD diagnosis was made 6 years after the birth of her son when her husband noticed drastic alterations in her mood. During a discussion about her mental health, she disclosed experiencing suicidal ideation and intrusive thoughts of harming her son, prompting her husband to transport GH to the emergency room for further evaluation immediately. After an assessment, she was prescribed medication and was released from the hospital, but she and her husband thought it best to relocate their son with her sister temporarily.

GH’s psychotropic medication history includes fluoxetine (Prozac), quetiapine (Seroquel), vilazodone (Viibryd), mirtazapine (Remeron), and alprazolam (Xanax). She reports that fluoxetine and alprazolam were prescribed for her PPD, and vilazodone, mirtazapine, and quetiapine were prescribed for her bipolar disorder. Fluoxetine was discontinued shortly after initiation due to headaches. Vilazodone was prescribed but stopped prematurely due to cost concerns before efficacy could be assessed. Mirtazapine was trialed without measurable symptom improvement. Quetiapine effectively reduced obsessive-compulsive symptoms related to orderliness and perfectionism but caused significant sedation, limiting her energy for perfectionistic tasks. While on quetiapine, GH adhered to an extended sleep schedule, retiring by 9:00 pm, waking briefly in the morning to take her children to school, and sleeping until the afternoon. Despite its sedative effects, she continued the medication to manage symptoms. GH also engaged in psychotherapy for 3 years following her OCD diagnosis but struggled to initiate exposure therapy due to anxiety about confronting her OCD symptoms.

At age 40, she decided to go on a KD for weight loss, during which she lost 50 pounds. Although she did not confirm ketosis with blood or urine samples, she noted the onset of a fruity and metallic taste in her mouth shortly after initiating the diet. After 1 year on the KD, GH experienced a complete cessation of her compulsive urges for orderliness, rule-following, and cleaning. The relief from her obsessions and perfectionistic tendencies marked the disappearance of the last of her OCD symptoms, which had previously persisted with psychotropic medication. Because of this newfound relief, she desired to taper off quetiapine to mitigate persistent sleepiness to enhance her ability to engage in more active daily routines. She believed that the medication had finally addressed her OCD and, as a result, felt it was no longer necessary to continue its use. Under the direction of her doctor, she tapered off quetiapine over 2 months. To her surprise, she was able to discontinue her medication safely and remained symptom-free. Unburdened by her symptoms of OCD and the side effects of her medication, she started regularly exercising, taking long walks, and doing yoga.

Four years after maintaining a strict diet adherence, her primary care physician tested her lipid profile, revealing a total cholesterol of 452 mg/dL, an LDL cholesterol (LDL-C) of 363 mg/dL, an HDL cholesterol (HDL-C) of 75 mg/dL, and triglycerides of 96 mg/dL. Concerned about these results, GH’s doctor advised adding starchy vegetables to her diet, starting with one sweet potato daily. Within 3 months, she experienced severe sleepiness and worsening depression. Reducing the intake to half a sweet potato did not help, and by 5 months, her lethargy and irritability intensified, with frequent outbursts. Her doctor then recommended a quarter sweet potato daily. By the seventh month, she had abandoned her exercise routine entirely. Soon after, obsessive-compulsive tendencies reappeared, with late-night cleaning driven by a compulsive need for order.

After experiencing a significant fluctuation in her mental health, which appeared to correspond with her adherence to a KD, GH chose to reinstate the diet following her relapse in symptoms. After 4 months, she became symptom-free again and returned to her exercise routines, reclaiming her energy and enthusiasm for life. GH has been in remission for 7 years, the latter six of which have been without any psychotropic medications. The patient’s Y-BOCS score was 21 before the initiation of the KD and decreased to 0 following the intervention. She primarily follows a carnivore diet, consuming beef, pork, and chicken. Occasionally, she consumes small amounts of berries, onions, and tomatoes, maintaining a low-carbohydrate approach on those days.

### Patient perspective

“Before eliminating sugars and grains from my diet, the best way to describe me was hollow, a shell of a person who could not fully engage in life. I feel like I missed the best part of my children’s young lives, sedated and overwhelmed by my thoughts. Now, I enjoy the person I am today—alive, active, and determined not to miss another second.”

### Timeline

**Figure 1:**
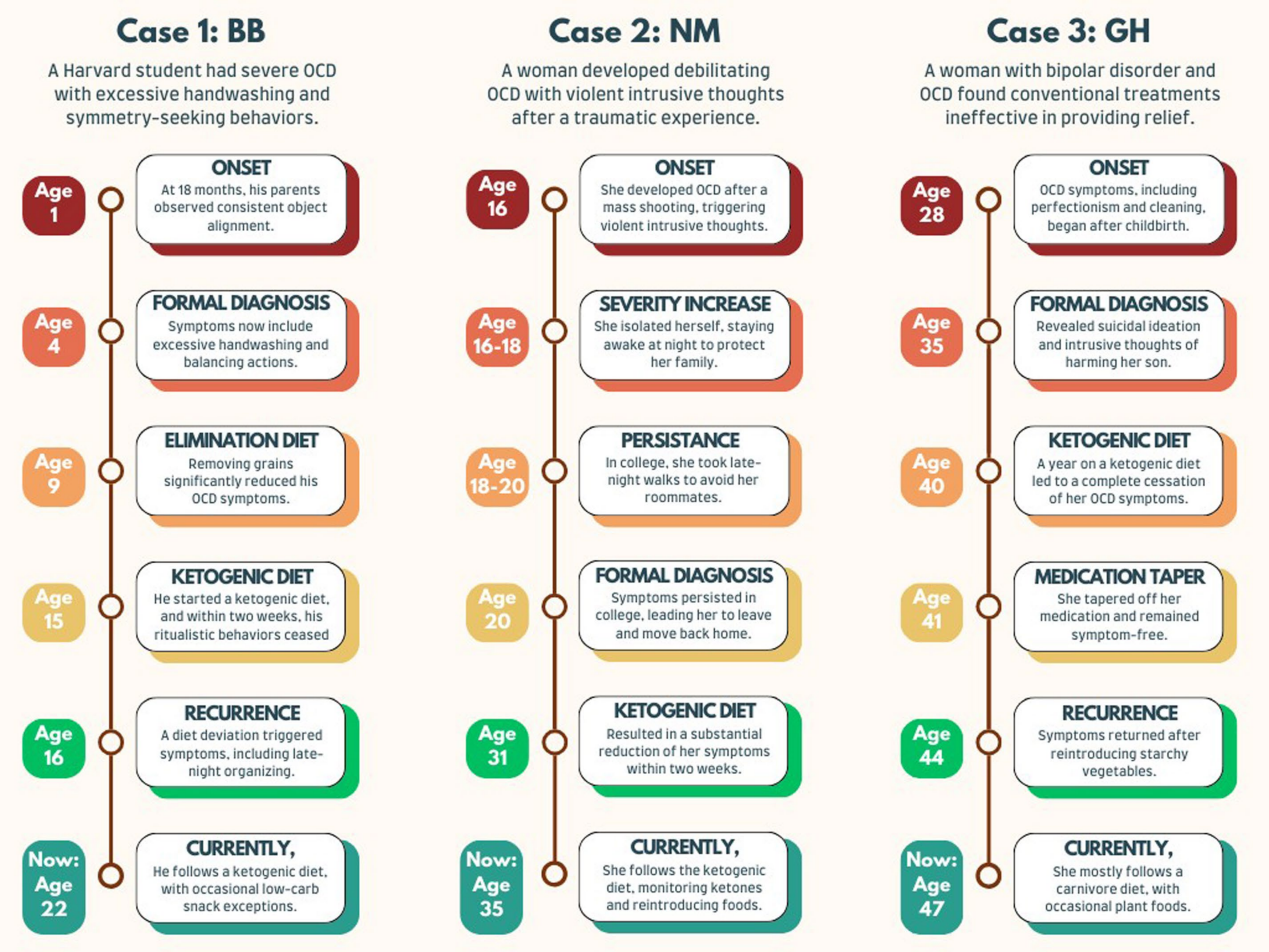
Treatment of obsessive-compulsive disorder with the ketogenic diet.

A chronological summary of the three case reports is illustrated in Figure 1.

## Discussion

### Strengths and limitations

These cases are suggestive of the KD’s potential as a treatment for OCD.

Although case reports alone do not provide sufficient evidence to fully assess treatment outcomes, it is important to highlight that all individuals in this study reported symptom recurrence following deviation from the KD, with a subsequent return of symptom relief upon re-adherence to the diet. This observation and the patients’ long-term commitment to a restrictive dietary regimen lend support to the validity of their reported experiences. None of the participants reported any other major life changes they felt contributed to their symptom remission.

While participants consistently reported symptom changes in response to dietary adherence or deviation, the lack of direct dietary monitoring or ketone blood tests prevents definitive confirmation of ketosis. This constraint limits the ability to quantitatively assess the precise metabolic effects underlying the reported symptom improvements.

Case series are generally prone to selection bias. The authors pursued and interviewed individuals who reported improvement in their OCD while on a KD. However, no candidates were rejected from the case series; the authors were committed to transparency, reporting participants’ stories without filtering for perceived significance or excluding less notable cases. Additionally, because Y-BOCS scores were obtained retrospectively, there is a potential for recall bias, as symptom severity was assessed from memory rather than through direct clinical evaluation.

Two of the participants in this report had not tried medication before implementing the KD. Because of this, it is uncertain whether traditional pharmaceutical interventions would have been effective for these individuals. This highlights the potential of the KD as an alternative therapeutic pathway and the importance of integrating it into a comprehensive treatment plan.

The patients in this study did not report any comorbidities or changes in their overall health after adopting the KD, other than improvements in their OCD symptoms and weight loss. Case 3, however, was diagnosed with Hashimoto’s thyroiditis in the years following adoption of the KD. It is uncertain whether the KD played a role in this diagnosis.

While this study does not establish causation, it offers a compelling perspective on addressing mental illness through metabolic pathways. Psychiatry, yearning for innovative solutions, faces a treatment-resistant rate of 20–60% among patients (18). Metabolic therapies could pave the way for transformative options, providing hope to those unresponsive to conventional psychotropic treatments.

### Concerns

Concerns about adopting a KD often center on its impact on lipid profiles and the perception that high-fat diets increase cardiovascular disease (CVD) risk. Implementing such a diet requires careful planning and a comprehensive risk–benefit evaluation tailored to the patient’s circumstances and goals.

A two-year randomized controlled trial with 322 moderately obese participants compared calorie-restricted low-fat (LFD), calorie-restricted Mediterranean (MD), and calorie-unrestricted low-carbohydrate diets (LCD) (19). The LCD achieved the greatest weight loss over that period and surpassed the MD and LFD in improving key CVD biomarkers, including HbA1c, triglycerides, HDL cholesterol, and C-reactive protein. These findings suggest that LCDs may mitigate CVD risk rather than exacerbate it.

### Direction of future research

Further research is needed to measure outcomes, ideally with randomized controlled trials with adequate participants. Ongoing research into the mechanisms underlying the KD’s effectiveness in treating other neuropsychiatric disorders may improve our understanding of its effects on OCD. Studies should include biomarker measurements such as the glutamate/GABA ratio, NAD+/NADH ratio, glucose metabolism, and/or inflammatory biomarkers to quantify alterations in metabolic dysfunction. Finally, ongoing research is needed to better elucidate the role of metabolic dysfunction in the pathophysiology of OCD.

## Conclusion

This study suggests that the KD may be an effective treatment for OCD. The participants in this study have sustained remission for years without the continuation of their medications. The KD appears to improve several pathophysiological processes associated with OCD and could target the underlying mechanisms of the disorder. Furthermore, the recurrence of symptoms upon dietary deviation underscores the KD’s therapeutic potential for neuropsychiatric conditions. Additional research into the effectiveness of the KD as a non-pharmaceutical treatment for OCD is warranted.

## Data Availability

The original contributions presented in the study are included in the article/Supplementary material, further inquiries can be directed to the corresponding authors.
