# Supplementary material for: Ketogenic diet as a therapeutic intervention for obsessive-compulsive disorder: a case series of three patients
Source: Front Nutr. 2025 Apr 16;12:1568076. doi: 10.3389/fnut.2025.1568076 (PMC12040614; doi:10.3389/fnut.2025.1568076)
Supplement: Supplementary file 2 [file Data_Sheet_2.PDF]

say this for a case study in 10 different ways: Before starting the ketogenic diet, the patient's Y-BOCS score was 22, which decreased to 3 after the intervention.

ChatGPT said:

ChatGPT

Here are 10 rephrased versions of the statement for the case study:

1. The patient’s Y-BOCS score was 22 prior to initiating the ketogenic diet and improved significantly to 3 following the intervention.
2. Before the ketogenic diet intervention, the patient had a Y-BOCS score of 22, which dropped to 3 afterward.
3. The patient’s Y-BOCS score, initially recorded at 22, decreased to 3 after implementing the ketogenic diet.
4. Prior to starting the ketogenic diet, the patient exhibited a Y-BOCS score of 22, which reduced to 3 post-intervention.
5. Following the ketogenic diet intervention, the patient’s Y-BOCS score improved from 22 to 3.
6. Initially, the patient had a Y-BOCS score of 22, which fell to 3 after adopting the ketogenic diet.
7. The ketogenic diet was associated with a reduction in the patient’s Y-BOCS score from 22 to 3.
8. The patient’s Y-BOCS score, which was 22 before starting the ketogenic diet, was reduced to 3 after the dietary change.
9. Before commencing the ketogenic diet, the patient’s Y-BOCS score was measured at 22 and later declined to 3.
10. The intervention led to a decrease in the patient’s Y-BOCS score, from 22 prior to the ketogenic diet to 3 afterward.

---

say this in 3 other ways for a case report: Finally, ongoing examination is needed in the intersection between OCD and metabolism.

ChatGPT said:

ChatGPT

Here are three alternative phrasings:

1. Continued exploration is essential to better understand the relationship between OCD and metabolism.
2. Further investigation into the connection between OCD and metabolic processes is warranted.

3. Ongoing research is necessary to delve deeper into the interplay between OCD and metabolism.

---

say this better for a case series abstract: The ketogenic diet is gaining traction as a therapeutic intervention for the treatment of neuropsychiatric disorders.

ChatGPT said:

ChatGPT

Here’s a more refined version for a case series abstract:

"The ketogenic diet is increasingly being explored as a potential therapeutic intervention for neuropsychiatric disorders."

This version maintains formality and clarity while emphasizing the emerging interest in the ketogenic diet as a treatment.

---

say 10 other ways to say this: professional connections and public outreach

ChatGPT said:

ChatGPT

Here are 10 alternative ways to say "professional connections and public outreach":

1. **Networking and community engagement**
2. **Industry relationships and public involvement**
3. **Professional networking and societal outreach**
4. **Collegial connections and public advocacy**
5. **Workplace networking and community outreach**
6. **Professional associations and public engagement**
7. **Career connections and civic outreach**
8. **Industry alliances and community interaction**
9. **Professional relationships and outreach initiatives**
10. **Business connections and public communication**

---

say this better for a case study: articipants were recruited through personal and professional connections made by one of the authors.

ChatGPT said:

ChatGPT

Here’s a refined version suitable for a case study:

"Participants were recruited through the personal and professional networks of one of the authors."

---

say this more formally for a case report: After he spoke to her about her depressed mood, she communicated to him that she was having thoughts of suicide, and he subsequently drove her to the emergency room for an evaluation.

ChatGPT said:

ChatGPT

After discussing her depressed mood with him, she disclosed experiencing suicidal thoughts, prompting him to transport her to the emergency department for further evaluation.

---

say this for a case reprot better: She describes feeling like walking on eggshells around her mother out of fear of initiating any conflict.

ChatGPT said:

ChatGPT

She reports feeling as though she is walking on eggshells around her mother, driven by a fear of provoking conflict.

---

say this better: This was occurring during treatment with medications and psychotherapy.

ChatGPT said:

ChatGPT

"This was happening concurrently with her treatment, which included both medication and psychotherapy."

---

say this formally: GH was first prescribed Prozac, but swiftly discontinued because it gave her headaches.

ChatGPT said:

ChatGPT

GH was initially prescribed fluoxetine; however, the medication was promptly discontinued due to the onset of headaches.

---

say this more professionally: While taking Seroquel, GH reports going to sleep around 9:00 pm, waking up to take her children to school, and going back to sleep, into the afternoon, until it was time to pick them up.

ChatGPT said:

ChatGPT

While taking Seroquel (quetiapine), GH reported adhering to a sleep schedule that involved retiring around 9:00 PM, waking briefly in the morning to take her children to school, and subsequently returning to sleep until the afternoon, when she would awaken to pick them up.

---

say this professionally: She participated in psychotherapy for three years, and while CBT was recommended, she struggled to begin exposure therapy due to anxiety surrounding the idea of routinely struggling with her OCD.

ChatGPT said:

ChatGPT

She engaged in psychotherapy for three years, during which cognitive-behavioral therapy (CBT) was recommended. However, she encountered difficulty initiating exposure therapy due to heightened anxiety regarding the prospect of consistently confronting her OCD symptoms.

---

say this more like th esleepiness was coming back and it was pervasive: Three months into this meal plan, she reports waking up in the morning, wishing she could go back to sleep

ChatGPT said:

ChatGPT

Three months into this dietary regimen, she began to experience a return of pervasive sleepiness, consistently waking up in the morning with a strong desire to return to sleep.

---

say this better for a case series: Encouraged by this unanticipated improvement, she has continued to adhere to the diet for the last three years, being entirely symptom-free.

ChatGPT said:

ChatGPT

Encouraged by this unexpected improvement, she has maintained adherence to the diet for the past three years and has remained completely symptom-free during this time.

---

say this professionally for a case series: NM disposed of all of her scarfs, belts, and any sharp objects in case the intrusive thoughts would manifest

ChatGPT said:

ChatGPT

NM removed all scarves, belts, and sharp objects from her environment to mitigate the perceived risk of acting on her intrusive thoughts.

---

say this better: Excessive neural activity as a measure of Glutamate/GABA imbalance has been observed in brain regions such as the anterior cingulate cortex (ACC) and CSTC circuitry.

ChatGPT said:

ChatGPT

Excessive neural activity, indicative of a glutamate/GABA imbalance, has been observed in key brain regions, including the anterior cingulate cortex (ACC) and the cortico-striato-thalamo-cortical (CSTC) circuitry.

---

say this better for a case report Interestingly, the KD has been shown to improve mitochondrial dysfunction, oxidative stress, inflammation, and glucose hypometabolism.

ChatGPT said:

ChatGPT

Interestingly, the KD has been found to alleviate mitochondrial dysfunction, reduce oxidative stress and inflammation, and mitigate glucose hypometabolism.

---

say this forally for a case reprot: Interestingly, the KD has been shown to improve mitochondrial function, decrease oxidative stress, inflammation, and glucose hypometabolism, and alleviate glutamate/GABA imbalances.

ChatGPT said:

ChatGPT

The ketogenic diet (KD) has been demonstrated to enhance mitochondrial function, reduce oxidative stress, mitigate inflammation, alleviate glucose hypometabolism, and restore the balance between glutamate and GABA, offering promising therapeutic effects in various neuropsychiatric conditions

mdpi

mdpi
.

---

say this very differently for a case report; We report on their experiences, discuss potential biological mechanisms for KD and ocd, highlight concerns about the diet, and make suggestions for the future direction of research.

ChatGPT said:
ChatGPT

This case report presents an analysis of their clinical outcomes, explores the biological mechanisms by which the ketogenic diet may influence obsessive-compulsive disorder, addresses potential limitations or risks associated with the diet, and proposes avenues for future investigation in this area.

---

say this part for the case series and make ti its own sentence and say it in passive voice: explores the biological mechanisms by which the ketogenic diet may influence obsessive-compulsive disorder, addresses potential limitations or risks associated with the diet, and proposes avenues for future investigation in this area.

ChatGPT said:
ChatGPT

The biological mechanisms through which the ketogenic diet may influence obsessive-compulsive disorder were explored, potential limitations or risks associated with the diet were addressed, and avenues for future investigation were proposed.

---

say this 10 differnt ways: All patients were shown a written version of the section of their report and asked to confirm details prior to consent.

ChatGPT said:
ChatGPT

Here are 10 different ways to phrase the sentence:

1. Each patient was provided with a written version of the relevant report section and asked to verify the information before giving consent.
2. A written version of the report section was presented to all patients, who were asked to confirm its accuracy prior to consenting.
3. All participants reviewed a written copy of their report section and were asked to confirm its details before providing consent.
4. The patients were shown a written excerpt from their report and instructed to verify the details before consenting.
5. A written copy of the relevant section of the report was given to each patient for confirmation prior to their consent.
6. Patients received a written version of the section of their report and were asked to verify its details before consent was obtained.
7. A written version of their report section was provided to all patients, who confirmed the details prior to consenting.
8. Patients were presented with a written version of the corresponding section of their report and asked to verify the information before providing their consent.
9. The patients reviewed and confirmed the details of a written version of their report section before consent was granted.
10. Each patient was given a written section of their report and asked to confirm the details before consent was given.

---

say this 10 different ways: Patients are presented with pseudo-initials to protect their identities.

ChatGPT said:

ChatGPT

Here are ten different ways to express that idea:

1. Patients are assigned pseudonyms to ensure their confidentiality.
2. To protect patient identities, pseudonyms are used instead of real names.
3. Pseudo-initials are provided to patients in order to safeguard their privacy.
4. The identities of patients are concealed by using fictional initials.
5. In order to maintain anonymity, patients are given alternative initials.
6. Patients' identities are anonymized through the use of pseudo-initials.
7. In efforts to protect patient confidentiality, fictional initials are assigned.
8. To preserve privacy, patients are referenced by pseudo-initials.
9. Pseudo-initials are employed to maintain the confidentiality of the patients.
10. Each patient’s identity is masked using substitute initials to ensure privacy.

These phrases communicate the same idea but use different wording to vary the expression.

---

say this better: They each sat for an interview and completed two Yale-Brown Obsessive Compulsive Scale (Y-BOCS) tests to evaluate the severity of OCD before starting the KD, and their current OCD.\

ChatGPT said:

ChatGPT

Each participant underwent an interview and completed two assessments using the Yale-Brown Obsessive Compulsive Scale (Y-BOCS) to assess the severity of their OCD prior to starting the ketogenic diet (KD) and to evaluate their current OCD symptoms.

---

say this better for a case series: Implementing a dietary regimen requires careful formulation, and a decision to intervene with any therapeutic diet should be made considering the patient's needs and priorities.

ChatGPT said:

ChatGPT

When implementing a dietary regimen, it is essential to carefully design the plan to meet the unique needs and priorities of the patient, ensuring that any decision to intervene with a therapeutic diet aligns with their specific circumstances and goals.

---

say this better for a case series: The continuing investigation into the mechanisms of how the KD functions to treat drug-resistant epilepsy may better our understanding of its effects on neuropsychiatric disorders.

ChatGPT said:

ChatGPT

Ongoing research into the mechanisms underlying the ketogenic diet's (KD) effectiveness in treating drug-resistant epilepsy could enhance our understanding of its potential benefits for neuropsychiatric disorders.

---

say this better: More research is needed to increase our understanding of the pathophysiologies associated with OCD.

ChatGPT said:

ChatGPT

Further investigation is essential to deepen our understanding of the underlying pathophysiologies associated with obsessive-compulsive disorder (OCD).

---

say this better for a case study: Each patient was given a copy of their report, and the accuracy of their respective report was confirmed before providing consent and subsequent submission.

ChatGPT said:

ChatGPT

Each patient was provided with a copy of their report, and the accuracy of the report was verified prior to obtaining their consent for submission.
